# Bioinformatic detection of E47, E2F1 and SREBP1 transcription factors as potential regulators of genes associated to acquisition of endometrial receptivity

**DOI:** 10.1186/1477-7827-9-14

**Published:** 2011-01-27

**Authors:** Alejandro Tapia, Cristian Vilos, Juan Carlos Marín, Horacio B Croxatto, Luigi Devoto

**Affiliations:** 1 Instituto de Investigaciones Materno Infantil (IDIMI), Facultad de Medicina, Universidad de Chile, Santiago, Chile; 2 Facultad de Química y Biología, Universidad de Santiago de Chile, Santiago, Chile; 3 Facultad de Ciencias, Universidad del Bio-Bío, Chillán, Chile; 4 Centro para el Desarrollo de la Nanociencia y la Nanotecnología (CEDENNA), Santiago, Chile; 5 Centro FONDAP de Estudios Moleculares de la Célula (CEMC), Santiago, Chile

## Abstract

**Background:**

The endometrium is a dynamic tissue whose changes are driven by the ovarian steroidal hormones. Its main function is to provide an adequate substrate for embryo implantation. Using microarray technology, several reports have provided the gene expression patterns of human endometrial tissue during the window of implantation. However it is required that biological connections be made across these genomic datasets to take full advantage of them. The objective of this work was to perform a research synthesis of available gene expression profiles related to acquisition of endometrial receptivity for embryo implantation, in order to gain insights into its molecular basis and regulation.

**Methods:**

Gene expression datasets were intersected to determine a consensus endometrial receptivity transcript list (CERTL). For this cluster of genes we determined their functional annotations using available web-based databases. In addition, promoter sequences were analyzed to identify putative transcription factor binding sites using bioinformatics tools and determined over-represented features.

**Results:**

We found 40 up- and 21 down-regulated transcripts in the CERTL. Those more consistently increased were C4BPA, SPP1, APOD, CD55, CFD, CLDN4, DKK1, ID4, IL15 and MAP3K5 whereas the more consistently decreased were OLFM1, CCNB1, CRABP2, EDN3, FGFR1, MSX1 and MSX2. Functional annotation of CERTL showed it was enriched with transcripts related to the immune response, complement activation and cell cycle regulation. Promoter sequence analysis of genes revealed that DNA binding sites for E47, E2F1 and SREBP1 transcription factors were the most consistently over-represented and in both up- and down-regulated genes during the window of implantation.

**Conclusions:**

Our research synthesis allowed organizing and mining high throughput data to explore endometrial receptivity and focus future research efforts on specific genes and pathways. The discovery of possible new transcription factors orchestrating the CERTL opens new alternatives for understanding gene expression regulation in uterine function.

## Background

The human endometrium is a complex tissue whose cyclic regulation is mainly driven by the changing pattern of the ovarian steroidal hormones estradiol (E_2_) and progesterone (P_4_) [1]. The main function of the endometrium is to provide receptive substrate at the appropriate time for blastocyst implantation. Although it is non-adhesive to embryos throughout most of the menstrual cycle [2] the action of P_4 _on an E_2_-primed endometrium induces a certain gene expression profile that is favorable for blastocyst adhesion during a restricted period of time known as the 'window of implantation' [3,4]. In women, this maternally directed receptive phase appears to be of approximately 5 days' duration, from day 20 to day 24 of a 28-days menstrual cycle [5]. The molecular basis of the window of implantation in human endometrium is beginning to be unrevealed and a number of biochemical markers for uterine receptivity have been proposed [3,6].

Microarrays analysis, an assay that is used to measure the level of mRNA expression of thousands of genes in a group of cells [7], enables discovery of genes or pathways likely to be involved in a biological process. This approach has been used to broadly characterize the molecular bases of endometrial function in women, by determining the gene expression profiles corresponding to each endometrial phase during the menstrual cycle [8-10]. In addition, it has been used to specifically investigate the acquisition of endometrial receptivity to embryo implantation during spontaneous cycles [11-15]. Since changes in the endometrium toward acquisition of receptivity are mainly driven by progesterone (P_4_) [16,17], two strategies have been used for gene discovery during spontaneous menstrual cycles. These are based on the comparison of the endometrial transcriptome under peak P_4 _circulating levels (days 19-23, window of implantation) compared to the endometrial gene expression profiles obtained under absent (days 8-11, proliferative phase) [11,12] or low (days 15-17, early secretory phase) [13-15,18,19] serum P_4_.

Although DNA microarrays are a powerful tool for gene discovery, there are several substantial sources of noise in microarray data. Intra- and inter-microarray variations limit the statistical power to discriminate the differentially expressed genes. While validation of microarray data is required to overcome this issue, most reports of endometrial gene expression analysis included validation of only a small number of differentially expressed genes (usually less than 10) by an independent mRNA quantification method (Northern blot, semi-quantitative or quantitative RT-PCR) [20]. Integration and cross-validation of data sets about endometrial gene expression profiles produced by different groups could increase confidence in gene expression results for many more genes than is tractable with classical validation [21,22] and should provide the up- and down-regulated genes that together orchestrate the acquisition of the receptive phenotype of the endometrium. Such exploration and integration could help researchers to obtain a comprehensive view of existing data and better prioritize experimental efforts.

Transcriptional regulatory mechanisms are crucial for temporal and spatial gene expression. These mechanisms are mediated by a set of transcription factors (TFs), proteins which have the ability to bind to a specific region on the gene (known as motifs or transcription factor binding sites (TFBS)), to regulate transcription. It is thought that co-expression of genes frequently arises from transcriptional co-regulation. As co-regulated genes share some similarities in their regulatory mechanism, possibly at transcriptional level, their promoter regions may contain common motifs that are binding sites for transcription regulators [23]. Given a cluster of endometrial regulated genes with similar expression profiles, the characterization of their regulatory regions is a fundamental step toward understanding the largely unexplored networks of gene regulation in this complex tissue responsible for their coordinated behavior. Computation biology of gene regulation offers several bioinformatic tools developed for the prediction of TFBS within a specific regulatory DNA sequence [24]. Given a set of co-regulated transcripts, *in silico *predictions of TFBS in their regulatory regions offers a unique opportunity to identify novel components, leading to the formulation of transcriptional regulatory networks hypotheses that can be further tested in the wet laboratory.

The aim of this study was to increase our understanding of endometrial receptivity to embryo implantation, by performing a research synthesis of the publicly available DNA microarray data. The first objective was to determine genes consistently reported in the literature as either up- or down-regulated from pre receptive to the receptive endometrium. The second objective was to identify possible TFs that may mediate the regulation of endometrial gene expression, by analyzing the *cis*-regulatory sequences of genes sharing a common regulatory behavior.

## Methods

### Integration and cross-validation of microarrays data

The available data sets comparing endometrial gene expression profiles from the proliferative vs. mid secretory phase [11,12] and from early secretory vs. mid secretory phase [9,13-15,19] were analyzed (Table 1). The UniGene key identifier (cluster ID) for each differential expressed transcript was obtained from the SOURCE [25], NetAffx [26] and UniGene [27] databases. Each UniGene entry is a set of transcript sequences that appear to come from the same transcription locus (gene or expressed pseudogene) and was used for cross-referencing transcripts amongst databases. The information from each database was imported into Microsoft Access^® ^software and used as a relational database to determine transcripts that show consistent differential expression under similar experimental conditions. Those having a similar transcriptional response (up- or down-regulation) in at least 4 reports for increased and 3 for decreased transcripts were considered biologically relevant and included in a list we have designated the 'consensus endometrial receptivity transcript list' (CERTL). The difference in threshold for considering down-regulated transcripts is because the study from Haouzi *et al *2009 [18] does not disclose the decreased transcripts.

**Table 1:** Endometrial gene expression reports performed at the time of implantation in human using DNA microarray

Study	First sample (day of cycle, number of samples)	Second sample (day of cycle, number of samples)	Microarrays platform	Fold-change cut-off value	N° of up-regulated transcripts	N° of down-regulated transcripts
Kao *et al. *(2002) [11]	Proliferative phase (8-11, n = 4)	Mid-secretory (21-23, n = 7)	Affymetrix Hu95A	≥2.0	156	377
Carson *et al. *(2002) [13]	Early-secretory (15-17, n = 3*)	Mid-secretory (20-22, n = 3*)	Affymetrix Hu95A	≥2.0	323	370
Borthwick *et al. *(2003) [12]	Proliferative phase (9-11, n = 5*)	Mid-secretory (19-21, n = 5*)	Affymetrix Hu95A-E	≥2.0	90	46
Riesewijk *et al. *(2003) [14]	Early-secretory (15, n = 5)	Mid-secretory (20, n = 5)	Affymetrix Hu95A	≥3.0	153	58
Mirkin *et al. *(2005) [15]	Early-secretory (16, n = 3)	Mid-secretory (21, n = 5)	Affymetrix HG_U95Av2	≥2.0	49	58
Talbi *et al. *(2006)^¶ ^[9]	Early-secretory (n = 3)	Mid-secretory (n = 8)	Affymetrix HG-U133 plus 2.0	≥1.5	1415	1463
Haouzi *et al. *(2009)^† ^[18]	Early-secretory (16, n = 31)	Mid-secretory (20, n = 31)	Affymetrix HG-U133 plus 2.0	≥2.0	945	67

* = pooled samples, ^¶ ^= day the cycle not specified, ^† ^= reports only the top 20 up-regulated genes as supplementary data, timing of endometrial biopsies based on first day of menses and not confirmed, possible endometrial pathologies were not excluded

### Functional clustering

Those up- and down-regulated genes from the CERTL were submitted to web-based databases for functional annotation analysis in order to gain an in-depth understanding of the biological themes in the CERTL. DAVID (Database for Annotation, Visualization and Integrated Discovery) [28] and GATHER (Gene Annotation Tool to Help Explain Relationships) [29] webtools were used for this purpose. Both services extract the biological meaning of submitted genes by retrieving their functional annotations from the Kyoto Encyclopedia of Genes and Genomes (KEGG) [30], Biocarta pathways [31] and Gene Ontology (GO) [32] databases.

### TFBS detection in promoter regions of genes associated to endometrial receptivity

We firstly examined the promoter region of our genes of interest defined as the region proximal to the transcription-start site of genes transcribed by RNA polymerase II. For a systematic search for potential TFBS, we used the following approaches and platforms to increase the power of our results:

MotifScanner. We used the stand-alone version of Motifscanner [33] that searches for potential TFBSs in a set of sequences using all the TRANSFAC vertebrate position-weigh matrices (PWMs) [34]. The information of TFBS obtained from MotifScanner was sent to the software TOUCAN [23] for determination of PWMs that were significantly over-represented.

Over-represented Transcription Factor Binding Site Prediction Tool (OTFBS). This web-tool [35,36] searches for potential TFBSs based on the TRANSFAC PWMs using the MatInspector algorithm [37] and determines over-represented motifs in regulatory sequences.

The Transcription Element Listening System (TELiS). The TELiS database [38,39] uses the TRANSFAC and JASPAR [40] PWMs in order to detect potential TFBS. It uses the MatInspector algorithm through the Java application PromoterScan [38] and identifies over-represented motifs.

GATHER. This database [29] searches for potential TFBS using TRANSFAC 8.2 PWMs [41,42] and identifies statically over-represented TFBS.

## Results

### Identification of genes associated to endometrial receptivity

We intersected the lists of regulated genes reported in studies using microarrays analysis of endometrial receptivity for determining those consistently regulated across different reports. As expected the number of coincident genes was small, considering the number of genes comprising each list. We identified 40 up-regulated genes in at least four of seven reports (Table 2) and 21 down-regulated genes present in at least three of six studies considered (Tables 3), collectively denominated CERTL. The most consistent up-regulated genes were C4BPA, SPP1, APOD, CD55, CFD, CLDN4, DKK1, ID4, IL15 and MAP3K5; whereas OLFM1, CCNB1, CRABP2, EDN3, FGFR1, MSX1 and MSX2 were the most consistently down-regulated in endometrial tissue for the acquisition of receptivity to embryo implantation.

**Table 2:** Up-regulated genes contained in the consensus endometrial receptivity transcripts list (CERTL) based on published reports about human endometrial receptivity using microarray analysis

UniGene ID	Gene Symbol	Gene Title	**Kao *et al. *(2002) **[11]	**Carson *et al. *(2002) **[13]	**Borthwick *et al. *(2003) **[12]	**Riesewijk *et al. *(2003) **[14]	**Mirkin *et al. *(2005) **[15]	**Talbi *et al. *(2006) **[9]	**Haouzi *et al. *(2009) **[18]
Hs.1012	C4BPA	complement component 4 binding protein, alpha	↑	↑	↑	↑		↑	↑
Hs.313	SPP1	secreted phosphoprotein 1 (osteopontin, bone sialoprotein I, early T-lymphocyte activation 1)	↑	↑	↑	↑	↑	↑	
Hs.522555	APOD	apolipoprotein D	↑	↑	↑	↑		↑	
Hs.126517	CD55	Decay accelerating factor for complement	↑		↑	↑	↑	↑	
Hs.155597	CFD	complement factor D (adipsin)	↑	↑	↑	↑		↑	
Hs.647036	CLDN4	claudin 4	↑	↑	↑	↑		↑	
Hs.40499	DKK1	dickkopf homolog 1 (Xenopus laevis)	↑	↑	↑	↑		↑	
Hs.519601	ID4	Inhibitor of DNA binding 4, dominant negative helix-loop-helix protein	↑	↑		↑	↑		↑
Hs.654378	IL15	interleukin 15	↑	↑		↑	↑	↑	
Hs.186486	MAP3K5	mitogen-activated protein kinase kinase kinase 5	↑	↑	↑	↑	↑		
Hs.511605	ANXA2	annexin A2	↑	↑		↑		↑	
Hs.422986	ANXA4	annexin A4	↑			↑	↑	↑	
Hs.524224	C1R	complement component 1, r subcomponent	↑		↑		↑	↑	
Hs.80409	GADD45A	growth arrest and DNA-damage-inducible, alpha	↑		↑	↑	↑		
Hs.386567	GBP2	guanylate binding protein 2, interferon-inducible		↑	↑	↑		↑	
Hs.183109	MAOA	monoamine oxidase A	↑		↑	↑	↑		
Hs.532325	PAEP	progestagen-associated endometrial protein (glycodelin)	↑		↑	↑			↑
Hs.384598	SERPING1	serpin peptidase inhibitor, clade G (C1 inhibitor), member 1, (angioedema, hereditary)			↑	↑	↑	↑	
Hs.1584	COMP	cartilage oligomeric matrix protein				↑		↑	↑
Hs.558314	CP	ceruloplasmin (ferroxidase)			↑	↑		↑	
Hs.368912	DPP4	dipeptidyl-peptidase 4 (CD26, adenosine deaminase complexing protein 2)				↑		↑	↑
Hs.446392	DYNLT3	Dynein, light chain, Tctex-type 3	↑		↑	↑			
Hs.198862	FBLN2	fibulin 2		↑		↑		↑	
Hs.433300	FCER1G	Fc fragment of IgE, high affinity I, receptor for; gamma polypeptide		↑		↑		↑	
Hs.432132	G0S2	G0/G1switch 2	↑		↑	↑			
Hs.2681	GAST	gastrin			↑	↑		↑	
Hs.616962	GDF15	growth differentiation factor 15	↑	↑	↑				
Hs.105806	GNLY	granulysin		↑		↑		↑	
Hs.386793	GPX3	glutathione peroxidase 3 (plasma)				↑		↑	↑
Hs.497636	LAMB3	laminin, beta 3	↑			↑		↑	
Hs.433391	MT1G	Metallothionein-IG	↑	↑	↑				
Hs.262857	PRUNE2	Prune homolog 2 (Drosophila)		↑	↑	↑			
Hs.50223	RBP4	retinol binding protein 4, plasma				↑		↑	↑
Hs.654444	S100A4	S100 calcium binding protein A4		↑		↑		↑	
Hs.2962	S100P	S100 calcium binding protein P				↑		↑	↑
Hs.517070	SLPI	secretory leukocyte peptidase inhibitor				↑	↑	↑	
Hs.517033	TGM2	transglutaminase 2 (C polypeptide, protein-glutamine-gamma-glutamyltransferase)				↑	↑	↑	
Hs.525607	TNFAIP2	tumor necrosis factor, alpha-induced protein 2				↑	↑	↑	
Hs.695930	VCAN	versican				↑	↑	↑	
Hs.2157	WAS	Wiskott-Aldrich syndrome (eczema-thrombocytopenia)	↑	↑		↑			

Up-ward arrows indicate up-regulation of the respective transcript.

**Table 3:** Down-regulated genes contained in the consensus endometrial receptivity transcripts list (CERTL) based on published reports about human endometrial receptivity using microarray analysis

UniGene ID	Gene Symbol	Gene Title	**Kao *et al. *(2002) **[11]	**Carson *et al. *(2002) **[13]	**Borthwick *et al. *(2003) **[12]	**Riesewijk *et al. *(2003) **[14]	**Mirkin *et al. *(2005) **[15]	**Talbi *et al. *(2006) **[9]
Hs.522484	OLFM1	olfactomedin 1	↓	↓	↓	↓		↓
Hs.23960	CCNB1	cyclin B1	↓	↓		↓		↓
Hs.405662	CRABP2	cellular retinoic acid binding protein 2	↓	↓	↓			↓
Hs.1408	EDN3	endothelin 3	↓	↓		↓		↓
Hs.264887	FGFR1	fibroblast growth factor receptor 1 (fms-related tyrosine kinase 2, Pfeiffer syndrome)	↓	↓	↓			↓
Hs.424414	MSX1	msh homeobox 1	↓			↓	↓	↓
Hs.89404	MSX2	msh homeobox 2	↓	↓		↓		↓
Hs.523852	CCND1	cyclin D1		↓			↓	↓
Hs.524947	CDC20	cell division cycle 20 homolog (S. cerevisiae)		↓		↓		↓
Hs.1594	CENPA	centromere protein A	↓	↓		↓		
Hs.83758	CKS2	CDC28 protein kinase regulatory subunit 2		↓			↓	↓
Hs.530904	CSRP2	cysteine and glycine-rich protein 2	↓			↓		↓
Hs.367725	GATA2	GATA binding protein 2	↓		↓		↓	
Hs.596913	HPGD	hydroxyprostaglandin dehydrogenase 15-(NAD)		↓		↓		↓
Hs.654504	IHH	Indian hedgehog homolog (Drosophila)		↓	↓			↓
Hs.438720	MCM7	Minichromosome maintenance complex component 7		↓	↓		↓	
Hs.75823	MLLT11	Myeloid/lymphoid or mixed-lineage leukemia (trithorax homolog, Drosophila); translocated to, 11	↓	↓	↓			
Hs.143751	MMP11	matrix metallopeptidase 11 (stromelysin 3)		↓	↓			↓
Hs.2256	MMP7	Matrix metalloproteinase 7	↓	↓	↓			
Hs.658169	SFRP4	secreted frizzled-related protein 4	↓	↓	↓			
Hs.182231	TRH	thyrotropin-releasing hormone		↓	↓	↓		

Down-ward arrows indicate down-regulation of the respective transcript.

### Functional associations of transcripts from CERTL

To gain further understanding of the potential functional roles of regulated transcripts present in CERTL we obtained the functional annotations from each gene and determined the enriched processes from two different web-based tools. The up-regulated transcript list was consistently enriched with transcripts related to the immune response and complement activation whereas the down-regulated transcript list was enriched with transcripts related to cell cycle regulation (Tables 4 and 5).

**Table 4:** Functional annotation clusters for up- and down-regulated transcripts from CERTL obtained through GATHER webtool

Database	Functional annotation	number of genes	p Value
*up-regulated transcripts*			
Gene Ontology	response to stimulus	16	<0.0001
Gene Ontology	response to biotic stimulus	12	<0.0001
**Gene Ontology**	**defense response**	**11**	**<0.0001**
**Gene Ontology**	**immune response**	**10**	**<0.0001**
Gene Ontology	response to stress	9	0.0001
**Gene Ontology**	**complement activation, classical pathway**	**4**	**<0.0001**
**Gene Ontology**	**complement activation**	**4**	**<0.0001**
**KEGG Pathway**	**Complement and coagulation cascades**	**4**	**0.0002**
*down-regulated transcripts*			
Gene Ontology	morphogenesis	10	0.0001
**Gene Ontology**	**cytokinesis**	**4**	**0.0001**
Gene Ontology	skeletal development	4	0.0001
Gene Ontology	development	12	0.0002
**KEGG Pathway**	**cell cycle**	**4**	**0.0002**

Enriched functional annotations found in GATHER (Table 4) and DAVID (table 5) appear in bolded style.

**Table 5:** Functional annotation clusters for up- and down-regulated transcripts from CERTL obtained through DAVID webtool

Database	Functional annotation	number of genes	p Value
*up-regulated transcripts*			
GOTERM_CC_FAT	extracellular region	22	<0.0001
SP_PIR_KEYWORDS	signal	22	<0.0001
UP_SEQ_FEATURE	signal peptide	22	<0.0001
**GOTERM_BP_FAT**	**defense response**	**9**	**<0.0001**
**GOTERM_BP_FAT**	**positive regulation of immune response**	**7**	**<0.0001**
**GOTERM_BP_FAT**	**inflammatory response**	**7**	**<0.0001**
**GOTERM_BP_FAT**	**immune effector process**	**6**	**<0.0001**
**GOTERM_BP_FAT**	**complement activation**	**5**	**<0.0001**
**GOTERM_BP_FAT**	**immunoglobulin mediated immune response**	**5**	**<0.0001**
**GOTERM_BP_FAT**	**lymphocyte mediated immunity**	**5**	**<0.0001**
**GOTERM_BP_FAT**	**activation of immune response**	**5**	**<0.0001**
**KEGG_PATHWAY**	**Complement and coagulation cascades**	**5**	**<0.0001**
**SP_PIR_KEYWORDS**	**complement pathway**	**4**	**<0.0001**
GOTERM_BP_FAT	response to steroid hormone stimulus	4	0.0068
GOTERM_BP_FAT	cell cycle	7	0.0038
*down-regulated transcripts*			
GOTERM_BP_FAT	response to steroid hormone stimulus	4	0.0068
**SP_PIR_KEYWORDS**	**cell cycle**	**6**	**0.00045**
**KEGG_PATHWAY**	**cell cycle**	**4**	**0.0038**
**GOTERM_BP_FAT**	**cell division**	**5**	**0.0028**
**GOTERM_BP_FAT**	**cell cycle**	**7**	**0.0038**
**GOTERM_BP_FAT**	**regulation of cell cycle**	**6**	**0.00047**
SP_PIR_KEYWORDS	developmental protein	6	0.0041
UP_SEQ_FEATURE	metal ion-binding site:Zinc 1	3	0.0053

Enriched functional annotations found in GATHER (Table 4) and DAVID (table 5) appear in bolded style.

### Identification of consensus sequences for TFBS sites of CERTL

We hypothesized that genes showing a common regulatory behavior may also share common regulatory mechanisms such as TFBSs in their respective promoter regions. To identify these possible common regulatory patterns that should be over-represented in the CERTL, we took advantage of several publicly available bioinformatics tools. The potential TFBS were detected in a first step, and then those statistically over-represented in our endometrial gene cluster were determined. The results are listed in Table 6 for up- and down-regulated transcripts respectively. Interestingly, DNA binding sites for E47, Sterol Regulatory Element Binding Protein 1 (SREBP1) and E2F1 were the most consistently over-represented and present in both up- and down-regulated transcripts. The number of increased genes with predicted TFBS for E2F1, SREBP1 and E47 was at least 20, 13 and 7 respectively in a total of 40. Of 21 decreased genes the number of transcripts with predicted TFBS was at least 14, 2 and 3 respectively. Other TFs over-represented were MEF2, FREAC2 and ARNT.

**Table 6:** Transcription factor binding sites (TFBS) over represented in up- and down- regulated genes from CERTL

Up-regulated genes				Down-regulated genes			
Tool for TFBS analysis	Transcription factor name	TFBS matrix	p Value	Tool for TFBS analysis	Transcription factor name	TFBS matrix	p Value
MotifScanner	**E47**	TRANSFAC	0.008	MotifScanner	**E47**	TRANSFAC	0.002
	**MEF2**	TRANSFAC	0.002		**SREBP1**	TRANSFAC	0.007
	**SREBP1**	TRANSFAC	0.007		**ARNT**	TRANSFAC	0.001
TELIS	PBX1	TRANSFAC	0.0001	TELIS	**ARNT**	TRANSFAC	<0.0001
	AP1	TRANSFAC	0.0005		HNF1	TRANSFAC	0.007
	EVI1	TRANSFAC	0.002		HNF-1	JASPAR	0.007
	SOX5	TRANSFAC	0.003	OTFBS	Hb	TRANSFAC	<0.0001
	Sox-5	JASPAR	0.0031		BR-C Z1	TRANSFAC	<0.0001
	Pbx1	JASPAR	0.007		BR-C Z4	TRANSFAC	0.004
	**FREAC-2**	JASPAR	0.007		HFH-2	TRANSFAC	<0.0001
	SOX-9	JASPAR	0.008		HFH-3	TRANSFAC	<0.0001
OTFBS	GCN4	TRANSFAC	0.001		FOXJ2	TRANSFAC	<0.0001
	CP2	TRANSFAC	0.007	GATHER	NFY	TRANSFAC	0.004
	Ik-2	TRANSFAC	<0.0001		**E2F1**	TRANSFAC	0.006
	Bcd	TRANSFAC	<0.0001		DEAF1	TRANSFAC	0.007
	ARP-1	TRANSFAC	<0.0001				
	**MEF-2**	TRANSFAC	0.005				
	cap	TRANSFAC	0.004				
	**E47**	TRANSFAC	0.002				
	**SREBP-1**	TRANSFAC	<0.0001				
GATHER	SRF	TRANSFAC	0.001				
	NRF2	TRANSFAC	0.002				
	**E2F1**	TRANSFAC	0.002				
	**FREAC2**	TRANSFAC	0.003				
	HEB	TRANSFAC	0.004				
	ELK1	TRANSFAC	0.005				

Transcription factors predicted by more than one analysis tool appear in bolded style.

## Discussion

Scientific knowledge of how endometrial receptivity is regulated is fundamental for the understanding of the mechanisms that govern embryonic implantation. The availability of public datasets related to global endometrial gene regulation during the acquisition of the receptive phenotype, provides a tool for the analysis of regulation of gene expression using bioinformatics tools. Using DNA microarrays analysis, several approaches have been used for determining the genes of uterine receptivity assessing the endometrium in different physiological [9,11-15,18,43], pathological [44-48] or intervened conditions [49,50]. We here analyzed seven reports of endometrial gene expression profiling during spontaneous cycles: Carson *et al. *[13], Kao *et al. *[11], Borthwick *et al. *[12], Riesewijk *et al. *[14], Mirkin *et al. *[15], Talbi *et al. *[9] and Haouzi *et al. *[18]. Since the number of endometrial samples analyzed in each of these studies was limited, the question arises as to whether the groups investigated were representative of the population. This is a major concern for any statistical analysis. Therefore we considered all studies together in a research synthesis to provide a larger sample size thus consolidating the selection of actual regulated transcripts in the endometrium. A first step was to associate probes and available annotations in the reports that belong to the same UniGene cluster (*i.e. *with same UniGene ID), and then proceed to further comparisons to identify common transcripts that are similarly regulated during the window of implantation. Previous partial analyses [15,43,51-53] found very few transcripts to be consistently regulated. In our study we found 61 transcripts regulated in the same direction in the endometrium during the window of implantation; 40 were up-regulated in at least 4 of 7 studies and 21 were down-regulated in at least 3 of 6 reports analyzed.

The relatively small number of consistently regulated transcripts identified could be explained by the differences in the study design, number of samples included and the methodology used for data analysis. However, other factors should be considered when interpreting gene expression analyses related to endometrial receptivity. Importantly, the reports included here, all used RNA extracted from whole endometrial biopsies, tissue that comprises a number of different cell types, including epithelial (luminal and glandular), stromal fibroblasts, endothelial cells, vascular smooth muscle cells and lymphoid cells. Hence the endometrial changes induced by E_2 _and P_4 _result from the differential response of each cell type to the same hormones. A clear example is the down regulation of the PR during the secretory phase in endometrial epithelial cells but not in the stromal compartment [54]. Microdissection of cell subpopulations (for example, with laser capture [55]) may disclose the actual gene expression profiles of each cell subpopulations within the tissue context. In addition, any biopsy sample may not represent the complete endometrium since microenvironments occur within this tissue. Nevertheless gene expression profiling of endometrial biopsies during the window of implantation is one of the most promising strategies for gene discovery related to uterine receptivity.

The intersection of gene lists performed in the present study showed that most consistently increased transcripts during the window of implantation were C4BPA, SPP1, APOD, CD55, CFD, CLDN4, DKK1, ID4, IL15 and MAP3K5 whereas OLFM1, CCNB1, CRABP2, EDN3, FGFR1, MSX1 and MSX2 were the most consistently decreased. However, correlation of transcript abundance change with changes in the corresponding protein, followed by functional testing of the biological effect of that protein, is necessary to confirm the biological significance of the microarray changes.

The functional annotations of up-regulated genes within the CERTL showed a significant association to the immune response and complement activation. Most of these genes belong to the innate immune system, which is the immunological first line of defense that provides an immediate response through its ability to distinguish between 'infectious non-self' and 'non-infectious self' [56]. Therefore, innate immunity regulation in the endometrium is of fundamental significance for establishing a microenvironment that will provide adequate tolerance to the implanting embryo [57]. Regarding complement system regulatory proteins, their possible roles and expression levels in the endometrium throughout the normal menstrual cycle have been reported [58-62]. Most of these studies show an increase of complement-regulatory molecules during the secretory phase in human endometrium [58,61,62] in line with the increased mRNA levels of the complement system molecules C4b-binding protein (C4BP) and adipsin (complement component factor D, CFD) from the CERTL. It is postulated that the complement system might be conferring immunity to the uterine cavity, defending it against bacterial infection. In this sense, C4BP may provide a protective role to the embryo where an increased expression of an inhibitor of complement system activation could reduce the chance of a misdirected complement attack to the embryo (which is considered as a semiallograft). Indeed, C4BPA transcript levels are abnormally decreased in the endometrium during the receptive phase in women with endometriosis [44,63], implantation failure [46] and unexplained recurrent abortion [64], suggesting it may have a role in embryo implantation. By contrast, adipsin may have a non-complement function in the female reproductive tract as suggested for other complement-molecules [60]. Adipsin is necessary for the production of oviduct-derived embryotrophic factor-3 (ETF-3) [65,66] which stimulates embryo development [67,68]. Thus up-regulation of adipsin in human endometrium may assist the embryo during the implantation process as shown for other chemokines in the endometrium [69].

Several down-regulated genes within CERTL are associated with cell cycle regulation, including cyclin B1 (CCNB1) the most consistently down-regulated gene. CCNB1 binds to p34 (cdc2) to form the mitosis-promoting factor during G2 phase [70,71]. In human secretory phase endometrium, CCNB1 is decreased compared to the proliferative phase [72,73] supporting the microarray data used to construct the CERTL. Moreover, in endometrial cell cultures, P_4 _decreases the expression of CCNB1, inhibits cell proliferation and induces apoptosis, suggesting that cyclin B1 may play an important role in proliferation and differentiation of the endometrial tissue under steroidal regulation.

Cellular retinol binding protein-2 (CRABP2) is a cytosolic protein that binds retinoic acid (RA) with high affinity [74]. The CRABP2 transcript has been reported to decrease from the proliferative to the secretory phase in human endometrium [75], which is in line with the microarrays reports used for constructing our CERTL. The physiological effects of RA are mediated by members of two families of nuclear receptors [76,77] and they all have been detected by immunohistochemistry in human endometrium throughout the phases of the menstrual cycle [78] in epithelial and stromal cells. The fact that CRABP2 decreases in human endometrium at the time of embryo implantation might suggest that RA signaling is required to be silenced, since it shuttles RA to the RA receptors in the cell nucleus [74,78]. In the mouse uterus, CRABP2 decreases around the time of embryo implantation [79] whereas P_4 _induces the expression of cyp26a1, the enzyme responsible for RA catabolism in mouse uterine epithelial cells [80,81]. Knock down of cyp26a1 in mouse uterus decreases embryo implantation rate [82]. In addition, in human secretory endometrium, cyp26a1 mRNA level is ~20 times higher than in the proliferative phase [83]. Since the action of RA is essential for endometrial stromal cell decidualization [79] silencing of RA signaling during the window of implantation might prevent precocious decidualization of stromal cells that could compromise endometrial receptivity.

The cytokine endothelin-3 (EDN3) and fibroblast growth factor receptor-1 (FGFR1) were among the transcripts consistently down-regulated in the endometrium during the window of implantation. There is abundant evidence showing that both endometrial receptivity and blastocyst implantation are regulated by cytokines and growth factors [84]. Immunoreactive pro-endothelin-3 has been described in human endometrium in luminal and glandular epithelia; however cycle-dependent regulation of this molecule is not clear [85]. Its action in the human endometrium is suggested to be in paracrine vasoactive control of the uterine vascular bed [86]. However this cytokine has many other functions such as proliferation and development of several cell types [87-90]. In the mouse oviduct, EDN3 signaling has been associated with the regulation of transcripts related to TGFβ, IL-10, and C/EBP [91]. Its functional role in the human endometrium and the effects of its down-regulation during the window of implantation has yet to be determined. FGFR1 and its ligand FGF-2 have also been described in human endometrium [92-95]. Immunoreactive FGFR1 and its transcript are significantly higher in proliferative that in secretory human endometrium [93,94] supporting the down-regulation of this transcript included in the CERTL. However, not all studies have reported such endometrial regulation [95]. FGF-2 promotes endometrial stromal proliferation [94,96] and ovarian steroid hormones modulate its synthesis and function in endometrial cells [96,97]. The functional relevance of FGFR1 down-regulation in endometrial receptivity remains to be elucidated.

With regard to the TFs present in the CERTL, we found the inhibitor of DNA binding 4 (ID4) up-regulated and MSX-1 and -2 down-regulated. In animal models, uterine MSX-1 and -2 are down-regulated by P_4 _[98] or during embryo implantation [99-101]. Constant expression of *Msx1 *in the infertile *Lif*-/- mice uterus further supports a role for MSX-1down-regulation in endometrial receptivity [100]. ID4 TF is a member of a family of inhibitor of DNA binding proteins (Id) that has been associated with cell proliferation and differentiation [102-105]. Its regulatory effect in human endometrium is unknown. Many other TFs associated with endometrial regulation [106-120] have provided insights into the molecular basis of gene regulation for endometrial function in response to sex steroid hormones. We reasoned that the cluster of regulated genes derived from microarray experiments related to endometrial receptivity (*i.e. *CERTL) would allow a different strategy for TF discovery, namely comparative promoter analysis. This is based on the hypothesis that genes showing a common regulatory behavior may also share common regulatory mechanisms such as TFBSs in their respective promoter regions. Interestingly, we found that E47, E2F1 and SREBP1 are common TFBSs for up- and down-regulated transcripts from CERTL so it is likely that they orchestrate the changes in transcript profile for endometrial receptivity. None of these three TFs have been described in normal human endometrium in the context of their regulation during the menstrual cycle, in response to steroidal hormones or a regulatory role on uterine function. However, there is no guarantee that the revealed TFBS are indeed functional in the context of regulatory regions, hence biological verification is required.

The E2F1 TF belongs to the E2F family [121] and displays properties of both an oncogene (induction of proliferation) and tumor suppressor (induction of apoptosis) [122,123]. E47 is a TF that belongs to the class I bHLH proteins, also known as E proteins [124] which form homo- or hetero-dimers and bind to specific DNA sequences [125]. Sterol regulatory element-binding protein 1 (SREBP1) is a membrane-bound TFs that belongs to a family of basic helix-loop-helix-leucine zipper (bHLHLZ) TFs [126]. Upon activation, SREBP1 translocates into the nucleus where it binds to sterol regulatory sites located in the promoter regions of genes involved in cholesterol homeostasis and transport [127,128] such as the steroidogenic acute regulatory protein (StAR), a key regulator of steroidogenesis [129]. Function of bHLH TFs such as E47 can be blocked by Inhibitor of DNA binding (Id) TFs [130,131]. In addition, SREBP1 as a member of bHLHLZ family, may also be subjected to regulation by Id proteins [132]. In the CERTL ID4 transcript was up-regulated in the receptive endometrium: as a consequence E47 and SREBP-1 TFs may be less available for binding to DNA in target sequences and direct co-regulated transcripts. Interestingly, the TF E2F1 is involved in the transcriptional control of *id4 *gene expression [133], supporting our bioinformatics findings of overrepresented TFBSs.

It is well known that P_4 _is essential for the establishment and maintenance of pregnancy in the women and in this sense the study of its actions in the uterus has been focused on changes in gene expression [134,135]. Responses to P_4 _in reproductive tissues occur by the activation of classical nuclear P_4 _receptors (PRA and B), which upon binding with their ligand, function as TFs regulating gene expression [136]. In addition, many transcriptional actions of P_4 _require interactions with corepressors and coactivators [137-139]. However, P_4 _may also act in the uterus through at least two families of nonclassical membrane progestin receptors [140,141]. Hence the genomic and non-genomic pathways may interact and integrate to ultimately affect endometrial gene expression. Interestingly, two of the endometrial transcripts more consistently up-regulated during the mid-secretory phase, APOD and SPP1, do not display progesterone response elements in their *cis-*regulatory sequences [12,15] suggesting that P_4 _induction is not directly mediated by the ligand-bound PR. Interestingly both APOD and SPP1 genes display TFBS for E2F1 in their upstream regulatory sequences. In breast cancer cells, P_4 _up-regulates the expression of E2F1 and hence indirectly affects transcription of classic E2F1 target genes [115]. Such regulation of E2F1 induced by progestins has been shown to be multimodal since ligand-bound PR can regulate its transcription directly but also indirectly through other molecules to achieve further progestin-mediated regulation of E2F1 expression [142]. Whether E2F1 along with E47 and SRBP1 are also mediating the P_4 _transcriptional regulation in the endometrium for acquisition of receptivity has yet to be determined.

Identification of the CERTL and the possible regulatory TFs in the present research synthesis should not be viewed as an end in itself. Their real value increases only as these results move through to biological validation, ranging from the numerical verification of expression levels with alternative techniques, to ascertaining the actual regulatory role of the TFs in the endometrial transcriptional networks. Finally, for several transcripts contained in the CERTL, biological knowledge is completely lacking in relation to endometrial physiology, so extensive research is required to better understand the mechanisms underlying endometrial receptivity.

## Conclusion

In conclusion, a CERTL comprised of 61 transcripts consistently regulated in human endometrium during the receptive period for embryo implantation has been identified in this study. These transcripts are mainly involved in immune response, complement activation and cell cycle regulation; suggesting that these biological process are associated with the acquisition of the receptive phenotype. Finally, TFBS for E47, SREBP1 and E2F1 were over-represented in the regulatory region of genes from CERTL, suggesting that they may be mediating the effects of the ovarian steroidal hormones in the endometrial transcriptional regulation. Biological validation of such bioinformatic predictions will shed light on the transcriptional networks associated to uterine receptivity for embryo implantation. Moreover, this knowledge can potentially be applied to improve fertility in infertile patients.

## Abbreviations

bHLH: basic helix-loop-helix transcription factors; bHLHLZ: basic helix-loop-helix-leucine zipper; C/EBP: CAAT/enhancer-binding proteins; C4BP: C4b-binding protein; CCNB1: cyclin B1; CERTL: consensus endometrial receptivity transcript list; CFD: complement component factor D; CLDN4: claudin-4; CRABP2: cellular retinol binding protein-2; DAVID: database for annotation, visualization and integrated discovery; DNA: deoxyribonucleic acid; E_2_, estradiol; EDN3: endothelin-3; EDNRB: endothelin receptor type B; ETF-3: oviduct-derived embryotrophic factor-3; FGF: fibroblast growth factor; FGFR1: fibroblast growth factor receptor-1; GATHER: gene annotation tool to help explain relationships; GO: gene ontology; Id: inhibitor of DNA binding proteins; ID4: inhibitor of DNA binding 4; IL-10: interleukin-10; KEGG: Kyoto encyclopedia of genes and genomes; LH: luteinizing hormone; LIF: leukemia inhibitory factor; mRNA: messenger ribonucleic acid; OTFBS: over-represented transcription factor binding site prediction tool; P_4_: progesterone; RA: retinoic acid; RT-PCR: reverse transcription-polymerase chain reaction; SRE: sterol regulatory element; SREBP1: sterol regulatory element binding protein 1; TELiS: the transcription element listening system; TF: transcription factor; TFBS: transcription factor binding site; TGFβ: transforming growth factor-β.

## Competing interests

The authors declare that they have no competing interests.

## Authors' contributions

AT conceived of the study, participated in its design, carried out the bioinformatic analyses and helped to draft the manuscript. CV participated in the creation and intersection of database. JCM participated in its design and data analysis. HBC participated in the coordination and helped to draft the manuscript. LD contributed to data analysis and helped to draft the manuscript. All authors read and approved the final manuscript.
